# The pulmonary sequalae in discharged patients with COVID-19: a short-term observational study

**DOI:** 10.1186/s12931-020-01385-1

**Published:** 2020-05-24

**Authors:** Dehan Liu, Wanshu Zhang, Feng Pan, Lin Li, Lian Yang, Dandan Zheng, Jiazheng Wang, Bo Liang

**Affiliations:** 1 Department of Radiology, Union Hospital, Tongji Medical College, Huazhong University of Science and Technology, Jiefang Avenue No.1277, Wuhan, 430022 China; 2 Hubei Province Key Laboratory of Molecular Imaging, Wuhan, 430022 China; 3 MSC Clinical & Technical Solutions, Philips Healthcare, Beijing, 100000 China

**Keywords:** COVID-19, CT, Follow-up, Discharge, Sequalae, GGO, Fibrous stripe

## Abstract

**Background:**

A cluster of patients with coronavirus disease 2019 (COVID-19) pneumonia were discharged from hospitals in Wuhan, China. We aimed to determine the cumulative percentage of complete radiological resolution at each time point, to explore the relevant affecting factors, and to describe the chest CT findings at different time points after hospital discharge.

**Methods:**

Patients with COVID-19 pneumonia confirmed by RT-PCR who were discharged consecutively from the hospital between 5 February 2020 and 10 March 2020 and who underwent serial chest CT scans on schedule were enrolled. The radiological characteristics of all patients were collected and analysed. The total CT score was the sum of non-GGO involvement determined at discharge. Afterwards, all patients underwent chest CT scans during the 1st, 2nd, and 3rd weeks after discharge. Imaging features and distributions were analysed across different time points.

**Results:**

A total of 149 patients who completed all CT scans were evaluated; there were 67 (45.0%) men and 82 (55.0%) women, with a median age of 43 years old (IQR 36–56). The cumulative percentage of complete radiological resolution was 8.1% (12 patients), 41.6% (62), 50.3% (75), and 53.0% (79) at discharge and during the 1st, 2nd, and 3rd weeks after discharge, respectively. Patients ≤44 years old showed a significantly higher cumulative percentage of complete radiological resolution than patients > 44 years old at the 3-week follow-up. The predominant patterns of abnormalities observed at discharge were ground-glass opacity (GGO) (125 [83.9%]), fibrous stripe (81 [54.4%]), and thickening of the adjacent pleura (33 [22.1%]). The positive count of GGO, fibrous stripe and thickening of the adjacent pleura gradually decreased, while GGO and fibrous stripe showed obvious resolution during the first week and the third week after discharge, respectively. “Tinted” sign and bronchovascular bundle distortion as two special features were discovered during the evolution.

**Conclusion:**

Lung lesions in COVID-19 pneumonia patients can be absorbed completely during short-term follow-up with no sequelae. Two weeks after discharge might be the optimal time point for early radiological estimation.

## Background

Coronavirus disease 2019 (COVID-19) has become a worldwide outbreak since the first report in December 2019 in Wuhan, China. By 6 April 2020, 1,210,956 infected patients and 67,594 fatalities had been reported [1], representing a high capability of transmission and a lethal feature. Previous studies have revealed the radiological features at different stages of the disease [2–4], but the radiological manifestations during the convalescence period are still unclear. Wu et al. reported that residual pulmonary lesions such as GGO and intralobular and interlobular septal thickening could be persistently observed years after recovery from severe acute respiratory syndrome (SARS) [5]. It has remained a concern to medical professionals and the public whether similar severe sequalae also exist for COVID-19. This observational study aims to provide the radiographic manifestations of COVID-19 in discharged patients with chest CT follow-up.

## Methods

This study was approved by the Ethics Committees of Union Hospital, Tongji Medical College, Huazhong University of Science and Technology, and it followed the 1964 Helsinki Declaration and its later amendments or comparable ethical standards.

### Definition and criteria

The diagnostic criteria of COVID-19 pneumonia followed the diagnosis and treatment protocols from the National Health Commission of the People’s Republic of China. The discharge criteria were as follows: 1. afebrile for more than 3 days; 2. respiratory symptoms significantly improved; 3. improvement in radiological abnormalities on chest radiography or CT; and 4. two consecutive negative COVID-19 nucleic acid tests detected at least 24 h apart [6]. Complete radiological resolution was defined as the absence of any chest radiographic abnormality potentially related to infection [7].

### Patients

A total of 149 discharged patients previously diagnosed with COVID-19 in this single centre from 5 February 2020 to 10 March 2020 underwent serial chest CT scans on schedule until complete radiological resolution, including 21 patients who were preliminarily reported in the previous study (3). However, in this study, more patients were involved for further exploration, and the same aim was not considered.

### Thin-section CT scan and radiological follow-up

Thin-section CT scanning was performed on a commercial multi-detector CT scanner (Philips Ingenuity Core128, Philips Medical Systems, Best, the Netherlands; SOMATOM Definition AS, Siemens Healthineers, Germany) with a single inspiratory phase. The tube voltage was set as 120 kVp with automatic tube current modulation. From the raw data, CT images were reconstructed with a matrix size of 512 × 512 as axial images (thickness of 1.5 mm and increment of 1.5 mm) with hybrid iterative reconstruction (iDose level 5, Philips Medical Systems, the Netherlands) or a pulmonary B70F kernel and a mediastinal B30f kernel (Siemens Healthineers, Germany).

Chest CT scans were regularly performed at different time points, including at discharge and during the 1st, 2nd, and 3rd weeks after discharge. The conventional CT score with residual non-ground-glass opacity was evaluated at the time of CT performed at discharge as for the baseline estimation (5 lobes, score 1–5 for each lobe, range, 0 none, 25 maximum). Each of the 5 lung lobes was visually scored from 0 to 5 as: 0, no involvement; 1, < 5% involvement; 2, 5–25% involvement; 3, 26–49% involvement; 4, 50–75% involvement; 5, > 75% involvement. The total CT score was the sum of the individual lobar scores and ranged from 0 (no involvement) to 25 (maximum involvement) [8]. In addition, radiological characteristics at different time points, including GGO, fibrous stripe, thickening of the adjacent pleura, bronchovascular bundle distortion and small pleural effusion, were collected and estimated. The GGO was defined as hazy area of increased attenuation without obscuration of the underlying vasculature [9–11]. If complete radiological resolution was reached, no further chest CT scan was performed.

All image analysis was performed using the institutional digital database system (Vue PACS, version 11.3.5.8902, Carestream Health, Canada) by two radiologists (B.L. and L.Y. who had 25 and 22 years of experience in thoracic radiology, respectively), and final scores were determined by consensus.

### Statistical analysis

Statistical analysis was performed using IBM SPSS Statistics Software (version 24; IBM, New York, USA). Quantitative data are presented as the median with interquartile range (IQR), and the counting data are presented as the percentage of the total unless otherwise specified. The cumulative percentage of the endpoints was calculated. The comparisons of counting data were evaluated using the Chi-square test. A *p*-value of less than 0.05 was defined as statistically significant.

## Results

### Basic characteristics

A total of 149 patients with a male to female ratio of 67:82 were included in the study. The average age was 43 years old (IQR 36–56). The most common initial symptoms were fever (83.4%) and cough (37.9%). Comorbidities of hypertension, diabetes, bronchial asthma, and coronary heart disease were reported in 11.1, 4.1, 2.8, and 2.1% of the patients, respectively. The median lymphocyte count (1.54 ×  10^9^/L, IQR 1.24–1.93, normal range: 1.1–3.2 × 10^9^/L) and D-dimer level (0.32 μg/ml, IQR 0.19–0.56, normal range: 0–0.5 μg/ml) were almost normal at discharge. The details are summarized in Table 1.

**Table 1:** Basic characteristics and laboratory examination for patients with COVID-19

	***n*** = 149.
**Gender**	
Male	67 (45.0%)
Female	82 (55.0%)
**Age (y)**	43 (36–56)
**Triage**^**a**^	
Pneumonia	142 (95.3%)
Severe pneumonia	7 (4.7%)
**The initial symptoms of onset**	
Fever	121 (83.4%)
Cough	55 (37.9%)
Fatigue	38 (26.2%)
Myalgia	24 (16.6%)
Dyspnea	15 (10.3%)
Diarrhea	9 (6.2%)
**Medical history**	
Hypertension	16 (11.0%)
Diabetes	6 (4.1%)
Bronchial asthma	4 (2.8%)
Coronary heart disease	3 (2.1%)
**Laboratory examinations at discharge**	
Lymphocyte count (× 10^9^ cells per L) (1.1–3.2)	1.54 (1.24–1.93)
D-dimer (0–0.5 μg/ml)	0.32 (0.19–0.56)
**CT score at discharge**^**b**^	1 (0–2)

^a^The clinical triage was based on the diagnosis and treatment protocols of pneumonia caused by a novel coronavirus (trial version 7)

^b^A semi-quantitative CT scoring system was used to estimate the pulmonary involvement of only non-GGO lesions (includes: fibrous stripe and mixed patterns)

### Cumulative percentage of complete radiological resolution at different time points

At discharge, 12 (8.1%) patients reached complete radiological resolution. After that, the cumulative percentage of complete radiological resolution was 41.6% (62 patients), 50.3% (75), and 53.0% (79) during the 1st, 2nd, and 3rd weeks after discharge, respectively (Table 2).

**Table 2:** The cumulative percentage of complete radiological resolution at different time points

	*n* = 149.
Chest CT at discharge	12 (8.1%)
The 1st CT follow-up	62 (41.6%)
The 2nd CT follow-up	75 (50.3%)
The 3rd CT follow-up	79 (53.0%)

### Factors associated with resolution

There was no significant difference in complete radiological resolution at the 3-week follow-up between males and females and between ≤1 and > 1 CT scores at discharge, while a significant difference was observed between groups with different ages (Table 3).

**Table 3:** Correlative factors with resolution

	Complete radiological resolution (n)		***p***-value^a^
	Yes	No	
**Age (y)**			
≤ 44	58	26	**< 0.001**
> 44	21	44	
**Gender**			
Male	39	28	0.251
Female	40	42	
**CT score at discharge**			
≤ 1	60	54	0.864
> 1	19	16	

^a^Chi-square test

### Dynamic chest CT features

**Fig. 1:**
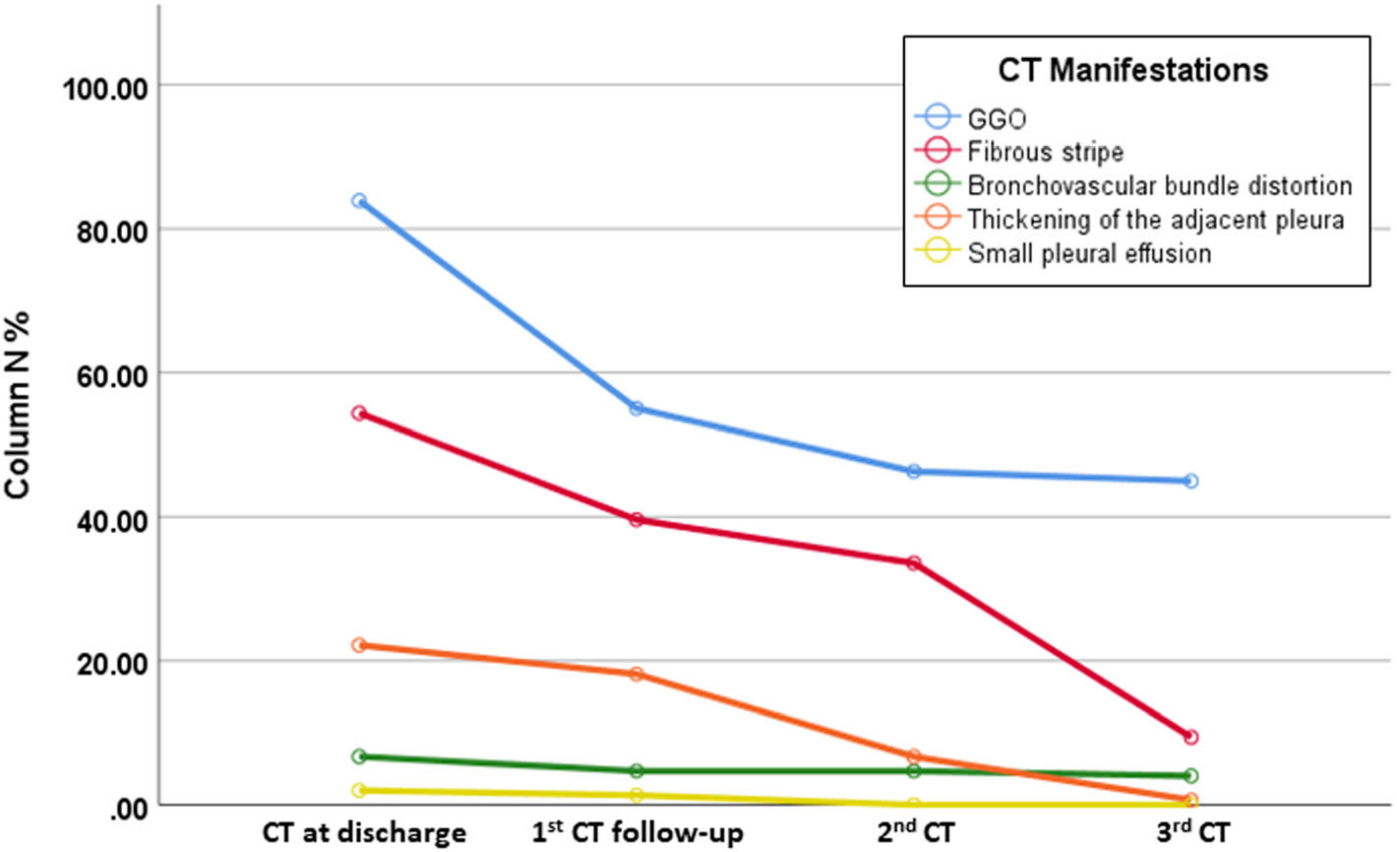
Dynamic changes of chest CT manifestation in different timepoint after discharged. Note: The predominant pattern were ground-glass opacity (GGO), fibrous stripe. With time, the positive count of GGO, fibrous stripe and thickening of the adjacent pleura gradually decreased, while GGO and fibrous stripe showed obvious resolution during the first week and the third week after discharge, respectively

**Fig. 2:**
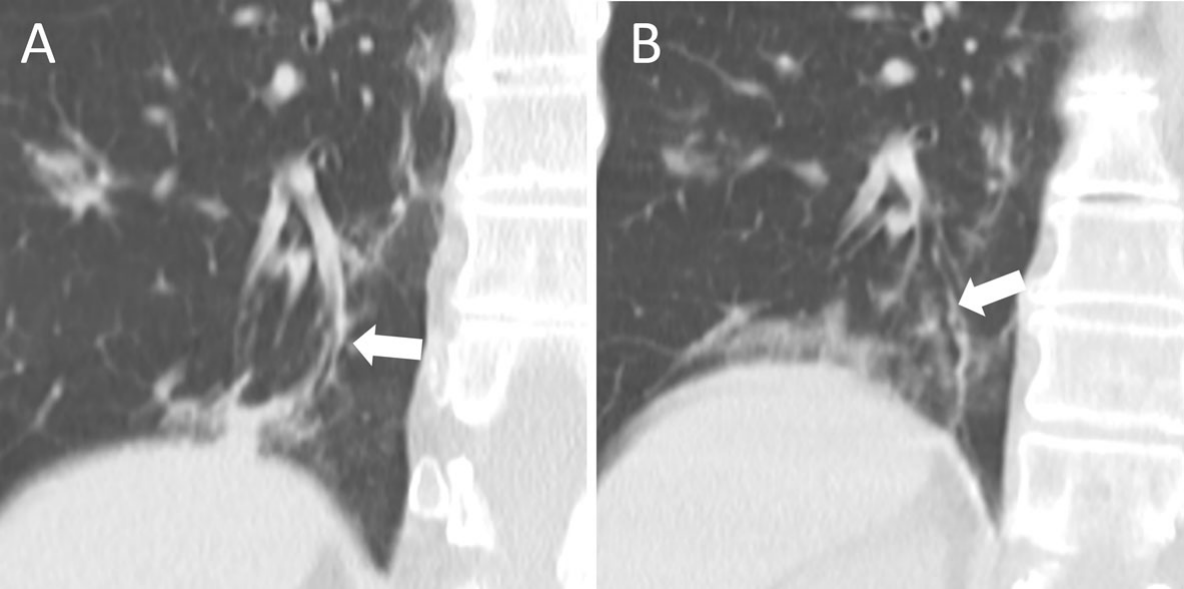
Coronal chest CT images of bronchovascular bundle of COVID-19 pneumonia. Note: (**a**). Bronchovascular bundle distortion; (**b**). Bronchovascular bundle distortion reversed. All images have the same window level of − 600 and window width of 1600

**Fig. 3:**
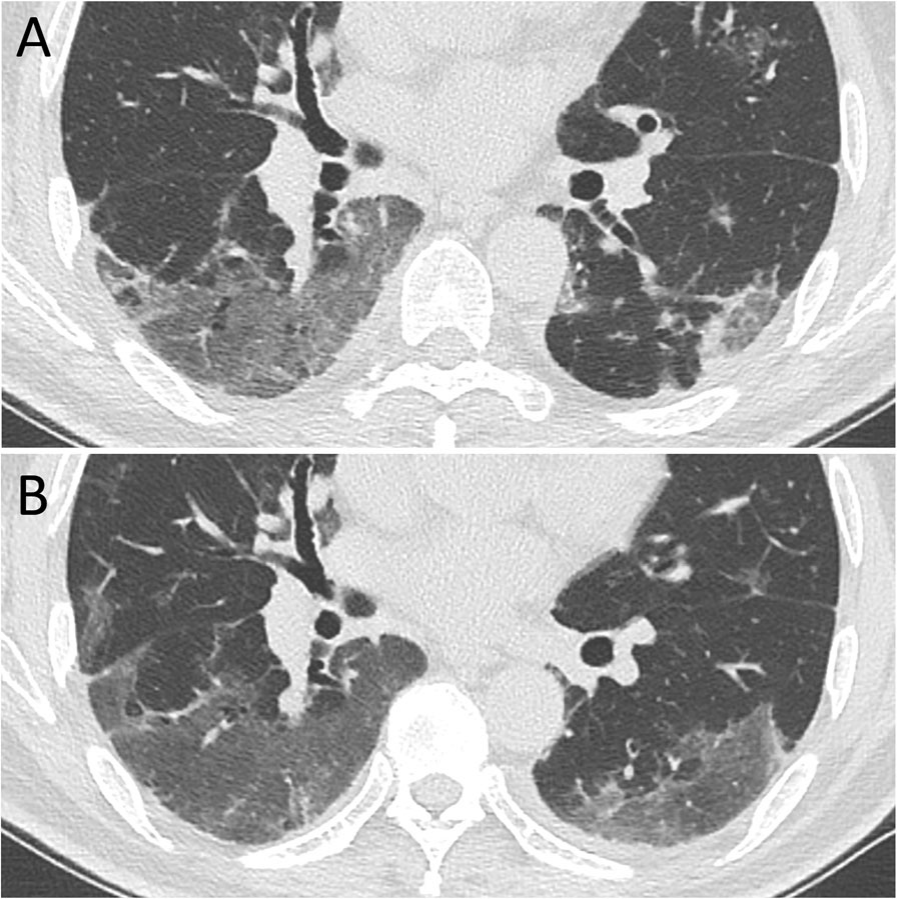
“Tinted” sign. Note: **(a).** GGO in lower lobes of both lung; (**b**). GGO faded out with a temporary extension of area after 7 days. All images have the same window level of − 600 and window width of 1600

The predominant patterns of abnormalities observed at discharge included GGO (125 [83.9%]), fibrous stripe (81 [54.4%]), and thickening of the adjacent pleura (33 [22.1%]) (Table 4). With time, the positive count of GGO, fibrous stripe and thickening of the adjacent pleura gradually decreased, while GGO and fibrous stripe showed obvious resolution during the first week and the third week after discharge, respectively (Fig. 1). Bronchovascular bundle distortion was found in 10 patients, with 4 instances of reversal (Fig. 2). GGO faded out with a temporary extension of area in 51 patients (Fig. 3).

**Table 4:** Chest CT manifestation in the short-term follow-up

CT manifestation	CT at discharge	1st CT follow-up	2nd CT follow-up	3rd CT follow-up
GGO	125 (83.9%)	82 (55.0%)	69 (46.3%)	67 (45.0%)
Fibrous stripe	81 (54.4%)	59 (39.6%)	50 (33.6%)	14 (9.4%)
Bronchovascular bundle distortion	10 (6.7%)	7 (4.7%)	7 (4.7%)	6 (4.0%)
Thickening of the adjacent pleura	33 (22.1%)	27 (18.1%)	10 (6.7%)	1 (0.7%)
Small pleural effusion	3 (2.0%)	2 (1.3%)	0 (0.0%)	0 (0.0%)

## Discussion

In this study, pulmonary lesions were found to be completely absorbed in 53.0% of patients during the 3rd week after discharge, implying that pulmonary damage caused by COVID-19 could be potentially repaired without any sequelae. However, more than 40% of patients demonstrated residual abnormalities, including GGO and fibrous stripe as the main CT manifestations at the 3-week radiological follow-up, for whom further radiological follow-up was continued. Two weeks was the appropriate median resolution period in this cohort.

The CT score of non-GGO lesions was used to evaluate residual pulmonary involvement. This was because an extended GGO area with decreased density may have occurred in some patients after discharge, and GGO is even a basic manifestation of convalescence, which could have led to over-estimation of the CT scores. As demonstrated in this study, patients with a CT score ≤ 1 at discharge did not show a faster resolution than patients with a CT score > 1, which indicated a stable baseline in the CT scores of all patients at discharge.

Previous studies have reported that older age and male sex are high risk factors for worse outcomes in patients with COVID-19 [12, 13]. However, in this study, under the milieu of similar residual pulmonary lesions in the patients at discharge, females did not show a faster resolution rate. On the other hand, younger age was associated with a better outcome in the convalescence period in this study, which is consistent with a former study [14].

**Fig. 4:**
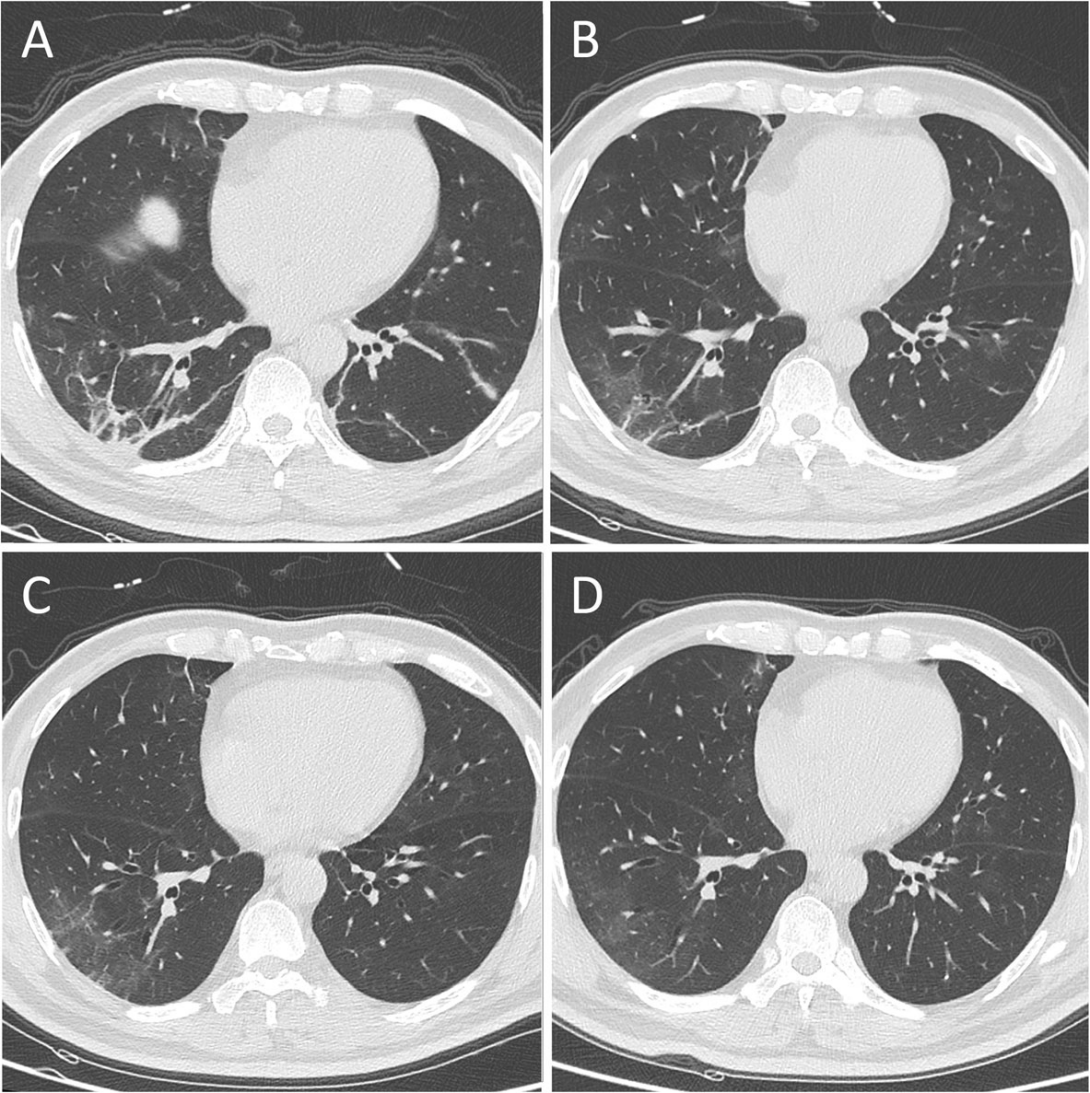
Dynamic resolution procedure of fibrous stripes. Note: 73 year-old male (**a**). Fibrous stripes showed in lower lobes of both lung (**b-d**). Fibrous stripes gradually absorbed with time. All images have the same window level of − 600 and window width of 1600

Radiological abnormalities have been reported to start resolving in the late phase in SARS, and the typical later-stage CT appearances were a coarse reticular pattern and GGO in the anterior part of the lungs [15]. For SARS, intralobular and interlobular septal thickening was observed to predominate over GGO even at 84 months [5]. In our cohort, GGO and fibrous stripe were the main imaging findings during the convalescence of COVID-19 pneumonia, which could be gradually absorbed completely, while a crazy-paving pattern was not demonstrated [3]. This observation addressed the question raised by Shi H et al. about whether the fibrosis in COVID-19 is irreversible [2]. There were three patterns in the residual lesions with proper evolution after discharge: 1. the extent of GGO was reduced and gradually faded until it completely disappeared; 2. fibrous stripes developed within the GGO area (mixed pattern), followed by gradual resorption and disappearance; and 3. fibrous stripes gradually reduced with decreasing density (Fig. 4). “Tinted” sign and bronchovascular bundle distortion as two special features were discovered during the evolution. The “tinted” sign was demonstrated as extension of the GGO area and a decrease in density, which might follow the “melting sugar” sign [16]. In this study, 51 patients were found with a “tinted” sign. The appearance of this pattern probably implies the gradual resolution of inflammation with re-expansion of alveoli and hence the resolution or recovery of illness, for which the pathological evidence merits further investigation. In the mixed pattern, 10 patients were found to have bronchovascular bundle distortion, with 4 patients with complete resolution during 17–37 days after discharge. This may be caused by inflammatory distraction or subsegmental atelectasis. In addition, 14 patients had varying extents of residual fibrous stripes, and the lesions were not fully resorbed at the end of the observation, which may be attributed to the limited follow-up time. The other CT manifestations in convalescence, such as thickening of the adjacent pleura and small pleural effusion, could also be absorbed completely.

A main limitation of the current study is that no critical patients were involved in this study, as they were still hospitalized in our centre at the end of the study. Second, only semi-quantitative estimation was carried out in this study for the complicated radiological characteristics in the convalescent stage.

In summary, this study showed the dynamic resolution process of lung lesions in discharged patients recovering from COVID-19. For most of the discharged patients, two weeks after discharge might be the optimal time point for early radiological estimation. Elderly patients need a longer time to reach complete radiological resolution. This study may help to understand the recovery course of this disease and indicate an optimized time point for chest CT scans in discharged patients.

## Data Availability

The datasets used and/or analysed during the current study are available from the corresponding author on reasonable request.

## Abbreviations

COVID-19: Coronavirus Disease 2019
SARS-CoV: Severe acute respiratory syndrome coronavirus
CT: Computed Tomography
IQR: Interquartile ranges
GGO: ground-glass opacity
RT-PCR: Reverse-transcription-polymerase chain-reaction

## References

[1] World Health Organization. Coronavirus disease 2019 (COVID-19) Situation report - 77 [https://www.who.int/docs/default-source/coronaviruse/situation-reports/20200406-sitrep-77-covid-19.pdf?sfvrsn=21d1e632_2. Published on April 6, 2020].

[2] Shi H, Han X, Jiang N, Cao Y, Alwalid O, Gu J, Fan Y, Zheng C (2020). Radiological findings from 81 patients with COVID-19 pneumonia in Wuhan, China: a descriptive study. Lancet Infect Dis.

[3] Pan F, Ye T, Sun P, Gui S, Liang B, Li L, Zheng D, Wang J, Hesketh RL, Yang L, Zheng C. Time Course of Lung Changes On Chest CT During Recovery From 2019 Novel Coronavirus (COVID-19) Pneumonia. Radiology. 2020:200370. 10.1148/radiol.2020200370 PMC7233367 32053470

[4] Wang Y, Dong C, Hu Y, Li C, Ren Q, Zhang X, Shi H, Zhou M. Temporal changes of CT findings in 90 patients with COVID-19 pneumonia: a longitudinal study. Radiology. 2020:200843. 10.1148/radiol.2020200843 PMC7233482 32191587

[5] Wu X, Dong D, Ma D (2016). Thin-section computed tomography manifestations during convalescence and long-term follow-up of patients with severe acute respiratory syndrome (SARS). Med Sci Monit.

[6] National Health Commission of the People’s Republic of China. Diagnosis and treatment protocols of pneumonia caused by a novel coronavirus (trial version 7) [http://www.gov.cn/zhengce/zhengceku/2020-03/04/content_5486705.htm. Published on March 3, 2020.].

[7] Bruns AH, Oosterheert JJ, El Moussaoui R, Opmeer BC, Hoepelman AI, Prins JM (2010). Pneumonia recovery: discrepancies in perspectives of the radiologist, physician and patient. J Gen Intern Med.

[8] Chang YC, Yu CJ, Chang SC, Galvin JR, Liu HM, Hsiao CH, Kuo PH, Chen KY, Franks TJ, Huang KM, Yang PC (2005). Pulmonary sequelae in convalescent patients after severe acute respiratory syndrome: evaluation with thin-section CT. Radiology.

[9] Franquet T (2011). Imaging of pulmonary viral pneumonia. Radiology.

[10] Koo HJ, Lim S, Choe J, Choi SH, Sung H, Do KH (2018). Radiographic and CT features of viral pneumonia. Radiographics.

[11] Hansell DM, Bankier AA, MacMahon H, McLoud TC, Muller NL, Remy J (2008). Fleischner society: glossary of terms for thoracic imaging. Radiology.

[12] Chen N, Zhou M, Dong X, Qu J, Gong F, Han Y, Qiu Y, Wang J, Liu Y, Wei Y (2020). Epidemiological and clinical characteristics of 99 cases of 2019 novel coronavirus pneumonia in Wuhan, China: a descriptive study. Lancet.

[13] Choi KW, Chau TN, Tsang O, Tso E, Chiu MC, Tong WL, Lee PO, Ng TK, Ng WF, Lee KC (2003). Outcomes and prognostic factors in 267 patients with severe acute respiratory syndrome in Hong Kong. Ann Intern Med.

[14] Opal SM, Girard TD, Ely EW (2005). The immunopathogenesis of sepsis in elderly patients. Clin Infect Dis.

[15] Sheard S, Rao P, Devaraj A (2012). Imaging of acute respiratory distress syndrome. Respir Care.

[16] Pan Y, Guan H, Zhou S, Wang Y, Li Q, Zhu T, Hu Q, Xia L. Initial CT findings and temporal changes in patients with the novel coronavirus pneumonia (2019-nCoV): a study of 63 patients in Wuhan, China. Eur Radiol. 2020. 10.1007/s00330-020-06731-x PMC7087663 32055945

